# Glyphosate Adsorption by Commercial Materials and Industrial Waste: Equilibrium and Kinetics

**DOI:** 10.1021/acsomega.5c10407

**Published:** 2026-03-18

**Authors:** Jéssica Piovesan Bertolo, Eduardo Dias Fenner, Jaqueline Steffler Leobett, Jonas Simon Dugatto, Janaina Silva Sarzi, Miqueias de Castro da Silva, Liziara da Costa Cabrera, Manuela Gomes Cardoso

**Affiliations:** † 665823 Universidade Federal da Fronteira Sul, Analytical Center Lab, Av. Jacob Reinaldo Haupenthal, 1580-Bairro São Pedro, 97900-000 Cerro Largo, Rio Grande do Sul, Brazil; ‡ Universidade Federal da Fronteira Sul, Multi-User Laboratory, Av. Jacob Reinaldo Haupenthal, 1580-Bairro São Pedro, 97900-000 Cerro Largo, Rio Grande do Sul, Brazil

## Abstract

Since glyphosate has been widely used in agriculture, it has frequently been detected in water bodies and has posed risks to environmental quality and human health. This study investigated glyphosate adsorption by commercial adsorbents (zeolite and activated carbon) and industrial residues (furnace slag, burning ashes, and foundry sand). Initial studies assessed the influence of pH (4, 7, and 10) and surface treatments with aqueous solutions of CuSO_4_, SDS, AgNO_3_, Fe­(NO_3_)_3_, CTAB, and ZnO on glyphosate removal. Among all materials and treatments, untreated burning ashes showed the highest removal efficiency and were selected for dosage, kinetic, and isotherm investigations. Glyphosate adsorption onto burning ashes was pH-insensitive and achieved 100% removal with the detection limit of 0.025 mg·L^–1^. In the dosage study (at an initial glyphosate concentration of 5 mg·L^–1^), 100% removal was reached when the ash dose was 25 g·L^–1^ and 87.91% when the dose was 1.25 g·L^–1^. The dose of 1.25 g·L^–1^ was defined as optimal not only because it met drinking water regulatory limits but also because it minimized material consumption. Regarding kinetics and equilibrium, some studies indicated that glyphosate adsorption equilibrium in ash was reached after 8 h with a maximum adsorption capacity of 5.39 mg·g^–1^. The Avrami kinetic model and the Temkin isotherm model exhibited the best fit to the experimental data. Glyphosate and AMPA were quantified by liquid chromatography-mass spectrometry (LC-MS) after derivatization. Results showed that burning ashes are capable of removing >95% of the initial concentration of up to 5 mg·L^–1^ glyphosate. Conclusions of this study indicate that the use of waste material is a promising and sustainable alternative for the removal of glyphosate from aqueous solutions.

## Graphical abstract

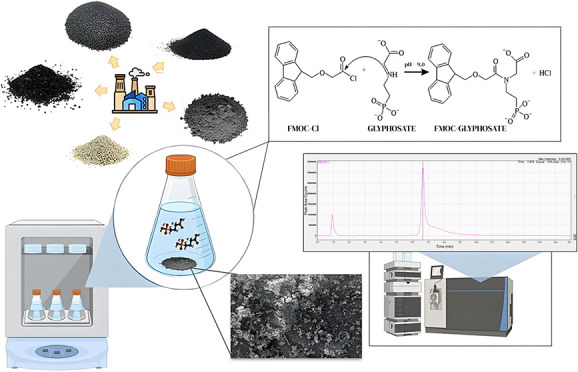

## 1 Introduction

Intensive agricultural practices, together with the growing demand for food around the world, have generated adverse environmental impacts.^1–3^ The use of large amounts of chemicals, such as herbicides, has been a common practice to increase crop productivity; however, it is also responsible for significant contamination of surface and groundwater.^4,5^ One of the most widely used herbicides is glyphosate, which has been intensively applied to grain crops, including genetically modified ones.^6,7^ A study revealed that approximately 880 tons of glyphosate were released into rivers around the world in 2020.^8^

Several regulations have addressed glyphosate and its metabolite AMPA (aminomethylphosphonic acid) in different countries. In Brazil, the Ministry of Health set the limit of 0.5 mg·L^–1^ for glyphosate + AMPA in water for human consumption,^9^ while, in the United States, Australia, and Canada, maximum limits are 0.7 mg·L^–1^,^10^ 1.0 mg·L^–1^,^11^ and 0.28 mg·L^–1^,^12^ respectively. The World Health Organization established that the maximum limit should be 0.9 mg·L^–1^.^13^

Some techniques, such as biofilters, photocatalysis and membrane filtration, which have its own characteristics and limitations, have been evaluated to enable glyphosate removal from contaminated water.^14,15^ Adsorption has been considered an alternative technique for glyphosate removal from aqueous systems due to its simplicity, versatility, and high efficiency. Recent studies have focused on some materials, such as biochar, zeolites, and activated carbons.^16–21^ Furthermore, chemical and physical treatments of adsorbents, such as modification with the use of surfactants and impregnation with metals, have been shown to be effective in increasing contaminant removal.^22–24^ The use of adsorption for contaminant removal allows waste valorization in the production of materials with adsorptive properties. It includes the use of biomass and industrial waste in the manufacture of activated carbons and zeolites and their direct application to pollutant removal processes.^25–28^

The foundry industry is important for the economy and production of metal parts for the automotive, aerospace, and agricultural sectors.^29^ Despite its economic relevance, the foundry industry has been associated with significant environmental challenges, such as harmful gas emission and solid waste generation. Its main waste is furnace slag, formed during metal melting with additives, sand discarded from molds used in the process and burning ashes composed of fine particles generated by furnaces and captured by bag filters.^30^ Due to their availability and physical stability, the materials represent an alternative for the adsorption of water contaminants.

Quantification of glyphosate in water poses significant challenges, since concentrations found in environmental samples are usually very low and require highly sensitive analytical techniques.^31^ The literature has described some methods of glyphosate detection in water which use the Liquid Chromatography coupled with Mass Spectrometry (LC-MS) technique, the most widely used one.^14^ However, the choice of the most appropriate analytical method depends on the specific characteristics of the sample and available resources in the laboratory.

Given the need to develop sustainable solutions to treat water contaminated with glyphosate, an alternative for using commercial materials and industrial waste for its adsorption has emerged. This study investigated glyphosate adsorption by commercial materials and industrial waste, focusing not only on the equilibrium and kinetics of the process but also on modifications with the use of surfactants and metallic salts.

## 2 Materials and Methods

### 2.1 Chemicals

Glyphosate (*N*-(phosphonomethyl)­glycine) (purity 96%), AMPA (purity 99%), 9-fluorenylmethoxycarbonyl chloride (FMOC-Cl) (purity 97%), acetonitrile (ACN) (Supelco), copper sulfate (CuSO_4_) (purity 97%), iron III nitrate (Fe­(NO_3_)_3_) (purity 99.7%), silver nitrate (AgNO_3_), zinc oxide (ZnO) (purity 99%), cetyltrimethylammonium bromide (CTAB) (purity 98%) and sodium dodecyl sulfate (SDS) (purity 90%) were purchased from Sigma-Aldrich. Ethyl acetate (Dynamics CAS 141–78–6) was purchased from Adonex. All reagents were HPLC grade. Milli-Q ultrapure water (Merck, Germany) was used for preparing the standards.

### 2.2 Glyphosate Determination

First, the determination of glyphosate involved the study of the derivatization procedure and, afterward, the chromatographic separation performed by a liquid chromatograph coupled with a mass spectrometer. Thus, several alternatives for glyphosate derivatization with FMOC-Cl were subject to a preliminary evaluation; different procedures adapted to the objective of this study and based on distinct methods were tested.^32,33^ The following aspects were evaluated: sample volume and conditioning, FMOC-Cl concentration, and borate buffer volume and concentration.

The initial evaluation led to the selection of the following method of derivatization: a 2 mL sample was adjusted to pH 9 by a borate buffer (0.4 mol·L^–1^). Then, 3 mL of FMOC-Cl (1 g·L^–1^ in ACN) was added. The mixture was stirred for 30 min and, to avoid photodegradation, a dark environment was created by wrapping the centrifuge tubes in aluminum foil. Subsequently, 3 mL ethyl acetate was added, followed by stirring for 3 min. Samples were subject to centrifugation at 3500 rpm for 4 min. The precipitate was filtered through a 0.22 μm membrane and transferred to polypropylene vials to undergo LC-MS analysis.

Chromatographic separation was performed by a liquid chromatograph coupled with a quadrupole mass spectrometer equipped with an electronebulization (ESI) ion source (Shimadzu). Mass/charge (*m*/*z*) ratios ranged from 390 to 168 (glyphosate) and from 332 to 110 (AMPA), due to formation of the derivative with FMOC-Cl and fragmentation during the mass spectrometric analysis. Table 1 summarizes the methodologies under evaluation.

**Table 1:** Analytical Methods Tested for LC-MS Analysis

parameter	option A	option B	option C	option D
column	C18 (2 μm × 2.0 × 100 mm)	C18 (2.7 μm × 3 mm × 50 mm)	ultra-amino (3 μm × 50 × 30 mm)	Zorbax HILIC plus (2.1 × 50 mm × 3.5 μm)
mobile phase	ultrapure water (A) and methanol (B), both with 5 mM ammonium formate	ultrapure water (A) and methanol (B), both with 5 mM ammonium formate	ammonium hydroxide (50 mM; pH 11) (A) and acetonitrile (B)	ultrapure water (A) and acetonitrile (B)
gradient	0.0–1.0 min 5% B; 7.0–9.0 min 100% B; 10–12 min 5% B	0.0–1.0 min 5% B; 7.0–9.0 min 100% B; 10–12 min 5% B	0.0–0.5 min 80% B, 0.5–2.5 min 40% de B, 2.5–4.5 min 20% de B, 5–8 min 80% de B	0.01–2.0 min 90% B; 10–13 min 5% B; 14–16 min 90% B
flow rate (mL min^–1^)	0.3	0.3	0.2	0.2
temperature	35 °C	35 °C	40 °C	40 °C
ionization mode	negative	positive	negative	negative
derivatization	yes	yes	no	yes
*m*/*z* (glyphosate)	390	390	168	390
*m*/*z* (AMPA)	332	332	110	332
refs	^32^	^32^	^34^	^35^

In the LC-MS analysis, the mass spectrometer was adjusted to the following tuning parameters: capillary voltage: −4.5 kV; detector voltage: 2.1 kV; interface temperature: 350 °C; desolvation line (DL) temperature: 250 °C; nebulizer gas flow: 1.5 L·min^–1^; heating block temperature: 200 °C; and drying gas flow: 15 L·min^–1^. Data processing was performed with LabSolutions Software (Shimadzu).

### 2.3 Adsorbent Materials

In this study, five materials were tested as potential glyphosate adsorbents: two commercial materials and three waste materials from a foundry located in the Rio Grande do Sul (RS) state, Brazil. They were clinoptilolite (Celta Brasil), sand (residual material from the molding process of metal parts), activated carbon (Dinâmica), furnace slag (residues from the metal melting process), and burning ashes (particulate material/dust retained in a bag filter).

In adsorption tests, residual materials were washed with distilled water and dried in an oven at 105 °C for 24 h. After drying, the blast furnace slag was ground to allow the disaggregation of particles and adequate weighing.

### 2.4 Treatment of Materials

The methodology of material treatment consisted in immersing the materials in functionalizing solutions with gentle agitation for a certain period.^36^ Functionalizing solutions were prepared using six chemicals: CuSO_4_, SDS, AgNO_3_, Fe­(NO_3_)_3_, CTAB, and ZnO, dissolved in water as described below.

In the treatment with CuSO_4_, SDS, AgNO_3_, and Fe­(NO_3_)_3_, a 50 mL aqueous solution was prepared at the mass/volume concentration of 8%. Regarding CTAB, a larger volume of water was needed for its dissolution, resulting in 125 mL of a 3.2% CTAB solution. To dissolve ZnO, 10 mL H_2_SO_4_ (6 mol·L^–1^) was added to 50 mL of the aqueous ZnO solution, resulting in 60 mL at 6.67% ZnO. After preparing the solutions, 10 g of every material was put in contact with the previously prepared solutions. The mixture was shaken on an orbital shaker table at 110 rpm and room temperature for 24 h. After stirring and filtration, materials were washed with distilled water and subsequently dried in an oven at 80 °C for 24 h, resulting in 30 different materials (Table 2).

**Table 2:** Mass/Volume Ratios of Adsorbent Materials and Functionalizing Solutions

	functionalizing solutions (mL)					
treatment	CuSO_4_ (8%)	SDS (8%)	AgNO_3_ (8%)	Fe(NO_3_)_3_ (8%)	CTAB (3,2%)	ZnO (6,67%)
dry materials (zeolite, sand, activated carbon, furnace slag, and burning ashes) (g)^a^	10 g	10 g	10 g	10 g	10 g	10 g
	50 mL	50 mL	50 mL	50 mL	125 mL	60 mL

a Every material was combined with different chemicals through immersion in functionalizing solutions. Thirty different materials were produced. Materials without any treatment were also evaluated to remove glyphosate.

### 2.5 Studies of Adsorption

Studies of glyphosate adsorption were carried out with the application of treated and untreated materials in a batch system. Herbicide solutions, in volumes of (40 mL each), were put in contact with the potentially adsorbent materials in polypropylene Erlenmeyer flasks, with masses ranging from 0.025 to 1 g. The system was agitated at 145 rpm in a Shaker incubator at 25 °C for 24 h. However, in the study of kinetics, which evaluated contact time, it ranged from 0.17 to 72 h. The solution pH, which ranged from 4 to 10, was also evaluated. Each experiment was performed in triplicate, and averages were used for the final data analysis.

Glyphosate concentration was quantified by LC-MS and the amount of adsorbed glyphosate was calculated by eq 1.

1 $$q=\frac{\left(C_{o}-C_{f}\right)\times V}{m}$$

Glyphosate removal was calculated by eq 2.

2 $$\%\mathrm{removal}=\frac{\left(C_{o}-C_{f}\right)\times 100}{C_{o}}$$

where: *q* is the amount of adsorbed glyphosate (mg·g^–1^); *C*_o_ is the initial concentration of glyphosate (mg·L^–1^); *C*_f_ is the final concentration of glyphosate (mg·L^–1^); *V* is the volume of the sample (L); *m* is the mass of the adsorbent (g).

### 2.6 Adsorption as a Function of Initial pH

In order to verify the influence of pH on glyphosate adsorption, tests were performed at pH 4, 7, and 10 with untreated materials. Hydrochloric acid (HCl) and sodium hydroxide (NaOH) solutions (0.1 mol·L^–1^) were used for adjusting the initial pH of the glyphosate solution (1 mg·L^–1^) containing 1 g of adsorbents.

### 2.7 Adsorption of Materials with and without Treatment

The study of the adsorption of materials was carried out with the use of 1 g of material and a glyphosate solution at an initial concentration of 1 mg·L^–1^. This study enabled the selection of a material and a treatment condition (with or without treatment) for the studies of dosage, kinetics, and equilibrium, and the characterization of the material. Chosen pH values were the ones that exhibited the best results in the previous study, i. e., pH 10 (zeolite), pH 4 (sand and activated carbon), and pH 7 (slag and burning ashes).

### 2.8 Studies of Dosage (Burning Ashes)

To study the effect of adsorbent dosage, different masses of untreated burning ashes were evaluated: 0.025; 0.05; 0.1; 0.2; 0.4; and 1 g, at pH 7. The selected pH and material were those that had the best results in studies of the initial pH and different treatments carried out in this study. The study of dosage was performed with a glyphosate solution at an initial concentration of 5 mg·L^–1^. The increase in the initial concentration from 1 mg·L^–1^ to 5 mg·L^–1^ was performed to allow a complete analysis since higher concentrations are more likely to saturate the adsorbent material and expose the real adsorption capacity of the system.

### 2.9 Studies of Kinetics (Burning Ashes)

In order to evaluate the adsorption rate over time, glyphosate solutions at pH 7 and an initial concentration of 5 mg·L^–1^ were put in contact with ashes and collected at time intervals of 0.17, 0.5, 1, 2, 4, 8, 16, 24, 36, 54, and 72 h. Kinetic data were analyzed by the pseudo-first-order model (eq 3), pseudo-second-order model (eq 4), Elovich model (eq 5), Weber-Morris intraparticle diffusion model (eq 6) and Avrami model (eq 7). The models were applied to fit the data by nonlinear fitting using the Scilab software.

3 $$q_{t}=q_{e}\times \left[1-\exp\left({-k}_{1}\times t\right)\right]$$

4 $$q_{t}=\frac{\left(q_{e}^{2}\times k_{2}\times t\right)}{\left(1+q_{e}\times k_{2}\times t\right)}$$

5 $$q_{t}=\frac{1}{\beta }\times \ln\left(1+\alpha \times \beta \times t\right)$$

6 $$q_{t}=k_{\mathrm{dif}}\times t^{0.5}+C$$

7 $$q_{t}=q_{e}\times \left(1-e^{-k_{\mathrm{av}}\times t^{n_{\mathrm{av}}}}\right)$$

where: *q_t_* is the amount adsorbed in time *t* (mg·g^–1^); *q*_e_ is the amount of adsorption at equilibrium (mg·g^–1^); *k*_1_ is the pseudo-first-order adsorption rate constant (min^–1^); *t* is time (min); *k*_2_ is the pseudo-second order adsorption rate constant (g·mg^–1^·min^–1^); α is the initial adsorption rate (mg·g·min^–1^); β represents the desorption constant (mg·g^–1^); *k*_dif_ is the intraparticle diffusion coefficient (mg·g^–1^·min^–0.5^); *C* is a constant related to the diffusion resistance (mg·g^–1^); *n*_av_ is the dimensionless Avrami number; *k*_av_ is the Avrami rate constant (min^–1^).

### 2.10 Studies of Equilibrium (Burning Ashes)

To determine the adsorption equilibrium isotherms, glyphosate solutions of 0.25, 0.5, 1, 1.5, 2.5, 5, and 7.5 mg·L^–1^ and 0.05 g burning ashes, determined as ideal by the study of dosage, were put in contact at pH 7 for 24 h. Equations proposed by Langmuir (eq 8), Freundlich (eq 9), BET (eq 10), and Temkin (eq 11) were used for fitting the models to the resulting experimental data. The isotherm models were applied to Solver and Scilab tools.

8 $$q_{e}=\frac{q_{e\max}\times K_{l}\times C_{e}}{1+K_{l}\times C_{e}}$$

9 $$q_{e}=K_{f}\times {C_{e}}^{1/n}$$

10 $$q_{e}=\frac{q_{\mathrm{BET}}\times K_{1}\times C_{e}}{\left(1-K_{2}\times C_{e}\right)\times \left(1-K_{2}\times C_{e}+K_{1}\times C_{e}\right)}$$

11 $$q_{e}=\frac{R\times T}{b}\times \ln\left(A\times C_{e}\right)$$

where: *q*_e_ is the amount adsorbed at equilibrium (mg·g^–1^); *q*_emax_ is the maximum adsorption capacity (mg·g^–1^); *K*_1_ is the Langmuir constant that determines the adsorption affinity (L·mg^–1^); *C*_e_ is the concentration of glyphosate after equilibrium (mg·L^–1^); *K*_f_ is the Freundlich capacity factor (mg·g^–1^ (mg·L^–1^)^−1/*n*^); *n* is the Freundlich intensity parameter; *q*_BET_ is the maximum adsorption capacity in multiple layers (mg·g^–1^); *K*_1_ is the equilibrium constant of the interaction in the monolayer (L·mg^–1^); *K*_2_ is the constant related to the saturation of the system; *A* is the Temkin constant related to the affinity of the adsorbent for the adsorbate (L·mg^–1^); *R* is the universal gas constant (8.314 J·mol^–1^·K^–1^); *T* is the absolute temperature (K); *b* is the constant related to the variation in adsorption energy (J·mol^–1^).

### 2.11 Model Adjustment and Analytical Determination

Analytical determination of the calibration curve was performed by the coefficient of determination (*r* ^2^) (eq 12) and the correlation coefficient (*r*) (eq 13). In the analysis of the adjustment of isotherm and kinetic models, the coefficient of determination (*r* ^2^) and the chi-square (χ^2^) (eq 14) were used.

12 $$r^{2}=1-\frac{\Sigma {\left(y_{i}-{\hat{y}}_{i}\right)}^{2}}{\Sigma {\left(y_{i}-{\mathrm{y̅}}_{i}\right)}^{2}}$$

13 $$r=\frac{\Sigma \left(x_{i}-\mathrm{x̲}\right)\left(y_{i}-\mathrm{y̅}\right)}{\sqrt{\Sigma {\left(x_{i}-\mathrm{x̲}\right)}^{2}}\Sigma {\left(y_{i}-\mathrm{y̅}\right)}^{2}}$$

14 $$\chi^{2}=\Sigma \frac{{\left(O_{i}-E_{i}\right)}^{2}}{E_{i}}$$

where: y*_i_* are observed values, ${\hat{y}}_{i}$ are values predicted by the model, ${\mathrm{y̅}}_{i}$ is the mean of observed values; *x_i_* and *y_i_* are values of the variables, *x* and y̅ are means of the variables; *O_i_* are observed values, *E_i_* is the expected value.

### 2.12 Characterization (Untreated Burning Ashes)

Transmittance analysis by Fourier transform infrared spectrometry (FTIR) was performed with the use of Shimadzu IRPrestige-21 equipment on KBr pellets (450–4000 nm). Scanning Electron Microscopy (SEM) images were taken by a Scanning Electron Microscope, in high and low vacuum modes, Jeol, JSM-6610LV. Elemental analysis was performed by energy dispersive X-ray spectroscopy (EDS) simultaneously with SEM imaging. Average pore size and surface area of burning ashes were determined by the Brunauer–Emmett–Teller (BET) isotherm at equilibrium time of 10 s and saturation pressure of 769.845 mmHg with N_2_. Determination of ζ-potential (ZP) was performed by the Brookhaven Instruments Zeta Plus equipment at a wavelength of 660 nm.

## 3 Results and Discussion

### 3.1 Glyphosate Determination

Chromatograms from the C_18_ column showed low selectivity; that is, intense signals of interferents occurred close to glyphosate retention time and affected separation. In addition, the sensitivity of the method was limited since it did not enable the detection of the analyte at low concentrations. Regarding the amino column, although the detection limit was similar to that of the HILIC column, its performance was affected by frequent clogging of the equipment. It occurred due to the presence of ammonium hydroxide in the mobile phase, which, not only caused obstructions in the system lines, but may also have interacted with the column components, thus, affecting stability and efficiency of the analysis. This problem made it difficult to continue the analyses and decreased reliability of the results. Therefore, the HILIC column (option D) was chosen as the ideal one for continuing the experiments. It enabled adequate analysis of glyphosate residues at detection limits that were suitable for the study and good selectivity, as shown by the chromatogram in Figure 1. However, to ensure good performance, more rigorous conditioning and cleaning of the column had to be performed for around 30 min to avoid interference and improve separation of the analyte.

**Figure 1:**
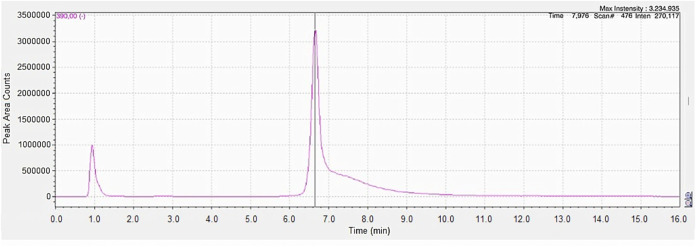
Glyphosate chromatogram.

In glyphosate quantification, analytical curves were prepared and injected into each batch of the experiment (Figure 2), maintaining linearity with a coefficient of determination (*r* ^2^) and correlation (*r*) above 0.9 at the concentration range from 0.025 to 7.5 mg·L^–1^. The lowest value on the curve was considered the limit of quantification (LQ of 0.025 mg·L^–1^). The limit of detection (LD) was considered to be 3 times lower than the LQ, i. e., 0.0083 mg·L^–1^.

**Figure 2:**
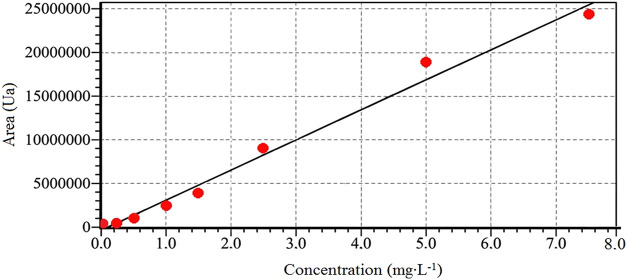
Analytical curve of the glyphosate standard.

In order to verify whether the initial commercial glyphosate solution was undergoing degradation over time, AMPA was monitored. The analysis was conducted under the same chromatographic conditions used for glyphosate (option D), maintaining consistent experimental settings. This approach allowed for precise monitoring of glyphosate degradation and AMPA formation and contributed to the evaluation of solution stability under the experimental conditions.

Results of the AMPA concentration were below the method detection limit of 0.0083 mg·L^–1^. The result demonstrates that AMPA is found at levels that are undetectable by the applied method. This condition may be associated with phenomena commonly observed in studies of adsorption, in which AMPA may be generated after slight degradation of glyphosate, or, alternatively, be removed and/or transformed through the action of the adsorbent material.

### 3.2 Characterization (Untreated Burning Ashes)

#### 3.2.1 FTIR

Spectra of burning ashes resulting from FTIR before and after adsorption are shown in Figure 3. Bands observed in the spectra were compared with observations made by studies of adsorption in aqueous media with the use of an adsorbent material whose composition was predominantly inorganic. Bands between 3400 and 3500 cm^–1^ are characteristic of the vibration of the hydroxyl group (−OH) of water molecules.^20,37^ The comparison before and after adsorption shows that an additional band appears in this range (before: 3563.64, 3491.31, and 3458.52 cm^–1^; after: 3564.60, 3485.52, 3459.48, and 3443.08 cm^–1^). It suggests that water may compete with glyphosate in interaction with burning ashes. The well-defined band at 1627.03 cm^–1^ is associated with vibrations of carbonyl groups (C=O).^28^ After adsorption, weakening of this band suggests a possible interaction between the adsorbate and adsorbent through this bond.

**Figure 3:**
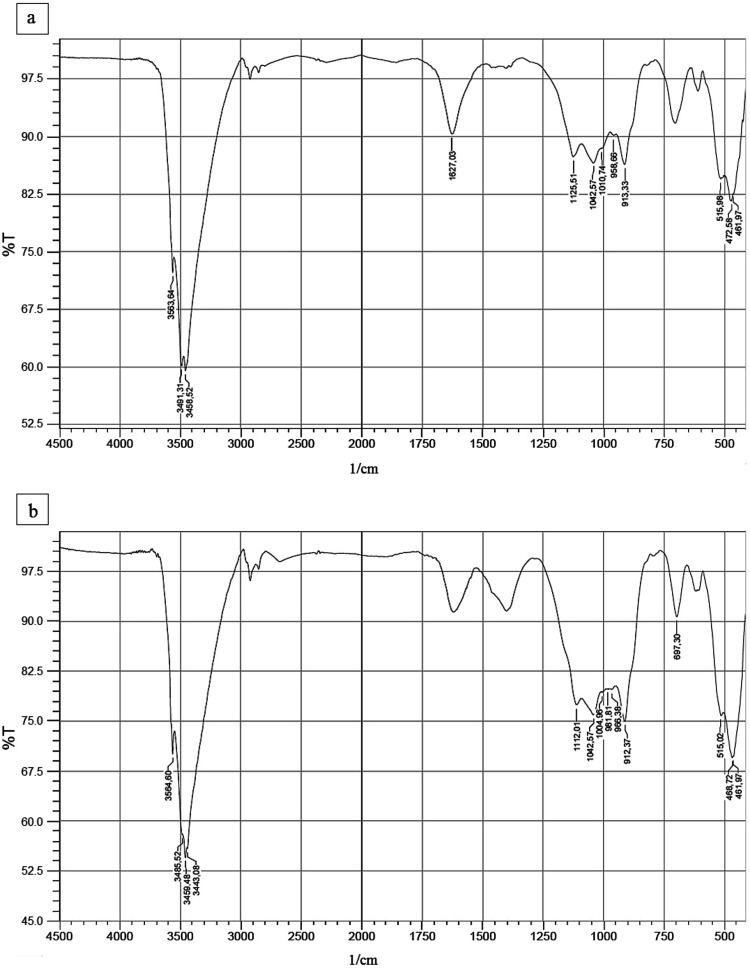
FTIR spectra of burning ashes before (a) and after (b) glyphosate adsorption. Captions: %T: % transmittance; 1/cm: wavenumber.

Bands between 913 and 980 cm^–1^ are generally associated with stretching or deformation vibrations in Si–O (silicon–oxygen) bonds in silicates, such as quartz (SiO_2_) and other silicon-containing minerals.^20^ The appearance of an additional band in the range again suggests the interaction of burning ashes with glyphosate. The well-defined bands at 1000 to 1125 cm^–1^ are typical of silicates, such as quartz (SiO_2_) and microcline (KAlSi_3_O_8_),^28^ while intense bands observed before and after adsorption, in the range from 515 to 475 cm^–1^, are characteristic of Fe–O vibrations and indicate the presence of iron oxides, such as magnetite and Fe_3_O_4_ in the material.^37^ The band observed at 697.30 cm^–1^ may be attributed to the elongation of Si–O–Al bonds.^20^ The presence of Si and Al in burning ashes was also indicated by the SEM-EDS analysis described below. Furthermore, intensification of this band after adsorption suggests chemical interaction between the herbicide and the oxides or silicates and confirms modifications in the material after the adsorption process.

#### 3.2.2 Scanning Electron Microscopy and Energy Dispersive Spectroscopy (SEM-EDS)

Images taken by SEM before and after the burning ashes adsorption process (Figure 4a,b) show differences in the surface of the material. In the postadsorption image, there are agglomerated particles (lumps) attached to the surface of the burning ashes. Besides, there is no uniformity in the geometry of particles on the surface, a common characteristic of residual materials.

**Figure 4:**
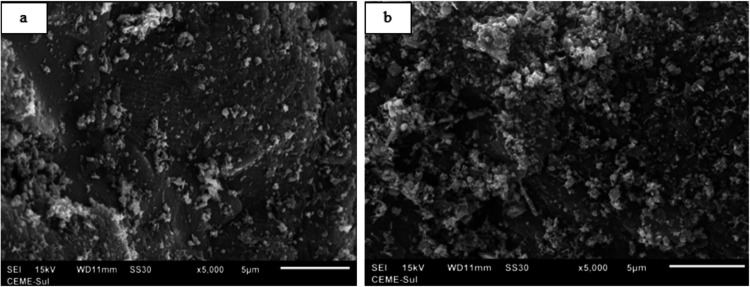
SEM images before (a) and after (b) the glyphosate adsorption process. At 5000× magnification, (b) shows an image that was taken after adsorption in a solution containing 5 mg·L^–1^ glyphosate.

The EDS was conducted to identify elements in the material surface and compare burning ashes before and after adsorption of 5 mg·L^–1^ glyphosate (Figure 5). Images after adsorption are divided between points 1 and 2. Results were displayed in the spectra of C, O, Si, Zn, Al, Au, Cl, Ca, and Fe in both adsorbents. The presence of C and Au is attributed to the materials used in the method of sample preparation, while the other elements are frequently found in minerals, such as aluminosilicates, clay minerals, and other types of soil. The EDS performed after adsorption revealed a low number of elements, since Mg, Mn and K are only found in the EDS before adsorption. This limitation in the detection of some compounds by EDS corroborates, with the difference observed in the images, suggesting that the surface of burned ash after adsorption was, in fact, covered by glyphosate.

**Figure 5:**
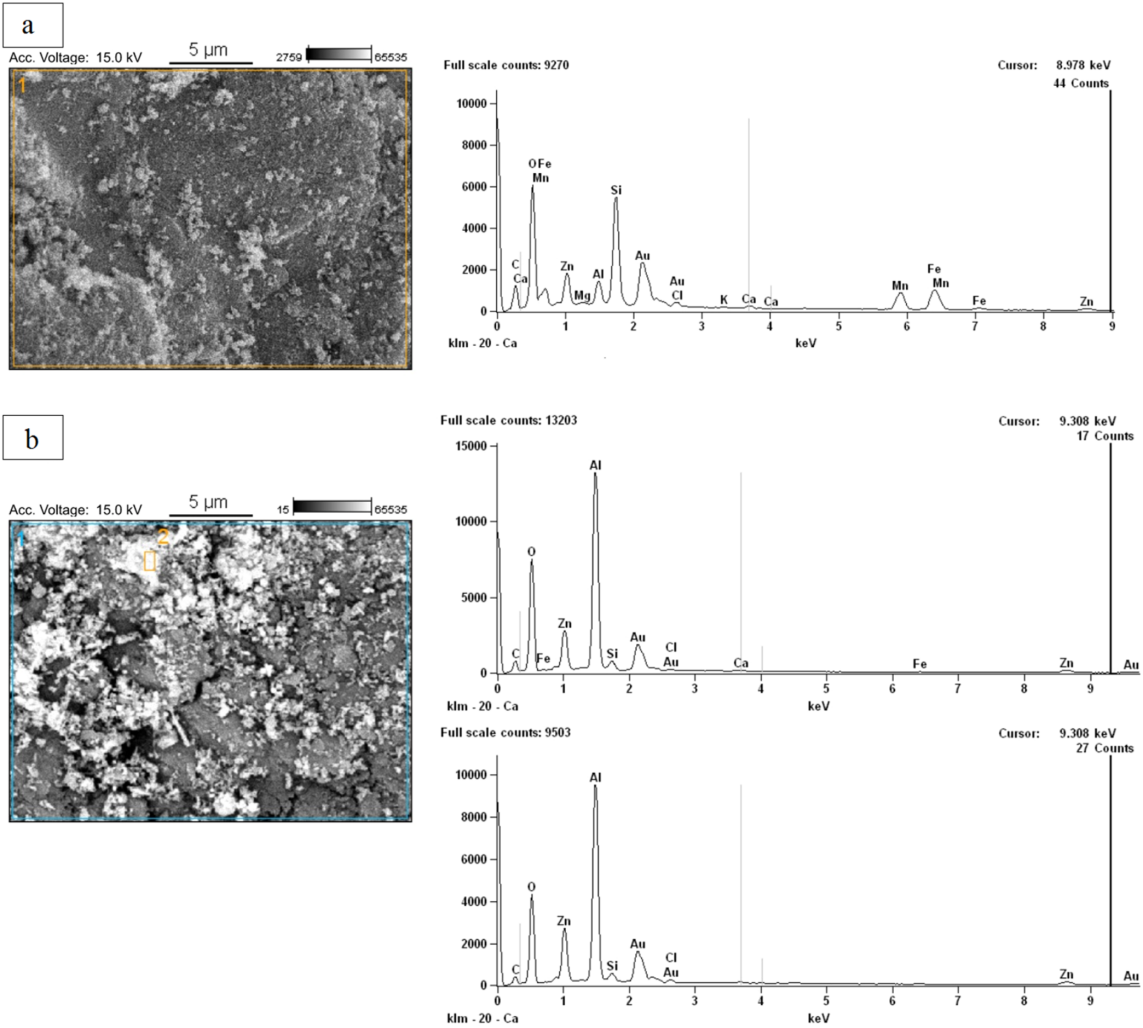
EDS images before (a) and after (b) the glyphosate adsorption process. At 5000× magnification, (b) shows an image that was taken after adsorption in a solution containing 5 mg·L^–1^ glyphosate.

#### 3.2.3 BET

A theoretical molecular size of glyphosate was estimated at approximately 0.8 nm,^38^ while the maximum radius projection of the molecule was 0.47 nm (diameter of 0.94 nm).^39^ Although the estimated molecular size is probably different from the real molecular size in dissolution and its zwitterionic behavior in the medium, it is smaller than the average width of pores of burning ashes (19.15 nm), a fact that indicates that the entry of glyphosate into the pores is assured if the geometry of the compounds is taken into account.

Pores may be classified according to their size into macropores (>500 Å), mesopores (200–500 Å), and micropores (<200 Å).^40^ Thus, according to the average pore width of burning ashes, they would be classified into adsorbents with microporous characteristics.

#### 3.2.4 ζ-Potential (ZP)

The ZP analysis of burning ashes resulted in −4.45 mV, indicating that the surface has a negative characteristic. It shows an unfavorable electrostatic affinity from the material with negatively charged compounds, such as glyphosate, which has a predominantly negative charge in its different dissociation states. However, the value found by this study is similar to the one found for calcite at pH between 2 and 6, which exhibited ζ-potentials between −4.1 mV and −3.5 mV,^41^ and has shown efficacy in glyphosate adsorption. Although the negative value suggests repulsion, compounds may be adsorbed by nonelectrostatic interactions, such as van der Waals forces and other mechanisms, which compensate for the electrostatic repulsion.

#### 3.2.5 Adsorption as a Function of Initial pH

pH is a parameter that affects the adsorption phenomenon. Figure 6 shows results of glyphosate removal (%) after 24 h of contact with different materials at every pH.

**Figure 6:**
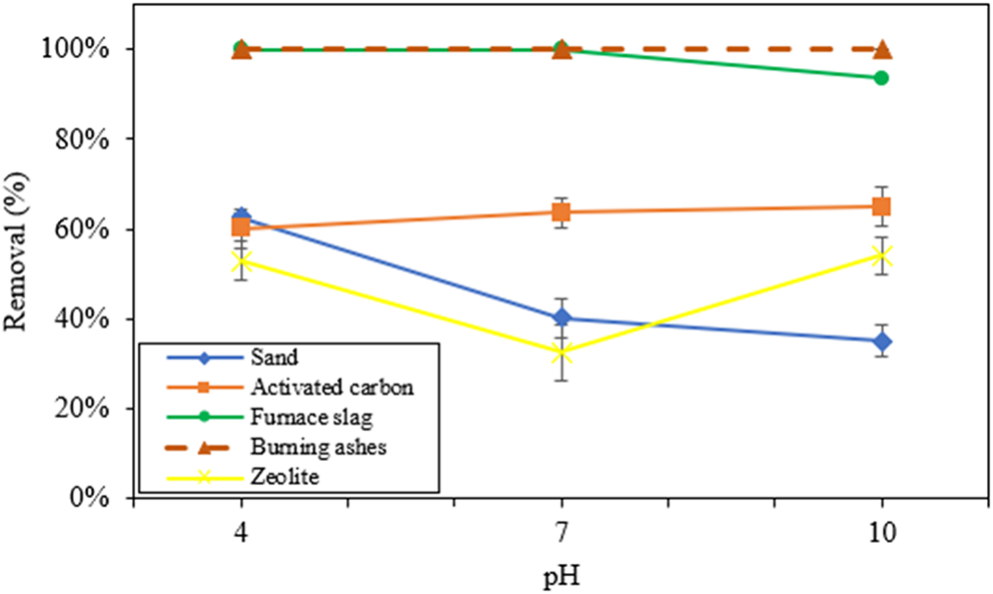
Removal (%) of glyphosate by untreated materials at different pH values, for a contact time of 24 h, at 145 rpm, m/v ratio of the solution of 25 g·L^–1^ (1 g adsorbent/40 mL glyphosate solution), at 25 °C and initial glyphosate concentration of 1 mg·L^–1^.

Table 3 shows values introduced by Figure 6, standard deviations, and final concentrations.

**Table 3:** Removal (%) of Glyphosate by Untreated Materials at Different pH Values, for a Contact Time of 24 h with Agitation, m/v Ratio of 25 g·L^–1^ Solution (1 g Adsorbent/0 mL Glyphosate Solution), at 25 °C and Initial Glyphosate Concentration of 1 mg·L^–1^ ^a^,^b^

pH	parameter	zeolite	sand	activated carbon	furnace slag	burning ashes
4	removal (%)	52.90	62.55	60.09	100^b^	100^b^
	Ce (mg·L^–1^)	0.222	0.103	0.342	0	0
	standard deviation	±0.04	±0.01	±0.04	±0	±0
7	removal (%)	32.38	40.17	63.65	100^b^	100^b^
	Ce (mg·L^–1^)	0.400	0.166	0.275	0	0
	standard deviation	±0.06	±0.04	±0.03	±0	±0
10	removal (%)	54.08	35.02	64.89	93.66	100^b^
	Ce (mg·L^–1^)	0.454	0.386	0.336	0.062	0
	standard deviation	±0.04	±0.03	±0.04	±0	±0

a Caption: Ce: Final concentration.

b Considering the quantification limit of the method of 0.025 mg·L^–1^

Results showed that the highest removal percentages were exhibited by burning ashes and furnace slag and that the values did not show any major changes in values of different pH under study. pH of the solution did not cause changes above 6.5% in removal with the use of activated carbon, furnace slag, and burning ashes, while in the cases of zeolite and sand, the pH altered the removal percentage by up to 20% (Figure 6). Concerning zeolite, sand, activated carbon, furnace slag, and burning ashes, pH values that resulted in the highest removal percentages were 10 (54.08%), 4 (62.55%), 10 (64.89%), 4 and 7 (100%), and 4, 7, and 10 (100%), respectively. The small variation found in removal percentages with pH may be based on the fact that the adsorbent material and glyphosate in solution change in a similar way to changes in pH.

To help the analysis of adsorption as a function of pH, it is helpful to observe the zwitterionic behavior of glyphosate (Figure 7), which changes in the molecular charge according to the pH of the medium. Based on the data for acid dissociation constants (p*K*_a_),^42^ we conclude that at high pH, glyphosate undergoes deprotonation and acquires a predominantly negative charge. At low pH, glyphosate protonates, which leads to a positive molecular charge. As indicated by the FTIR analysis, burning ashes also have ligands that may undergo changes as a function of the pH of the solution, such as hydroxyl groups −OH. Since no significant difference was found in the adsorption values of burning ashes, it is believed that although the pH of the medium alters the species, it may cause changes at the same intensity in both species.

**Figure 7:**
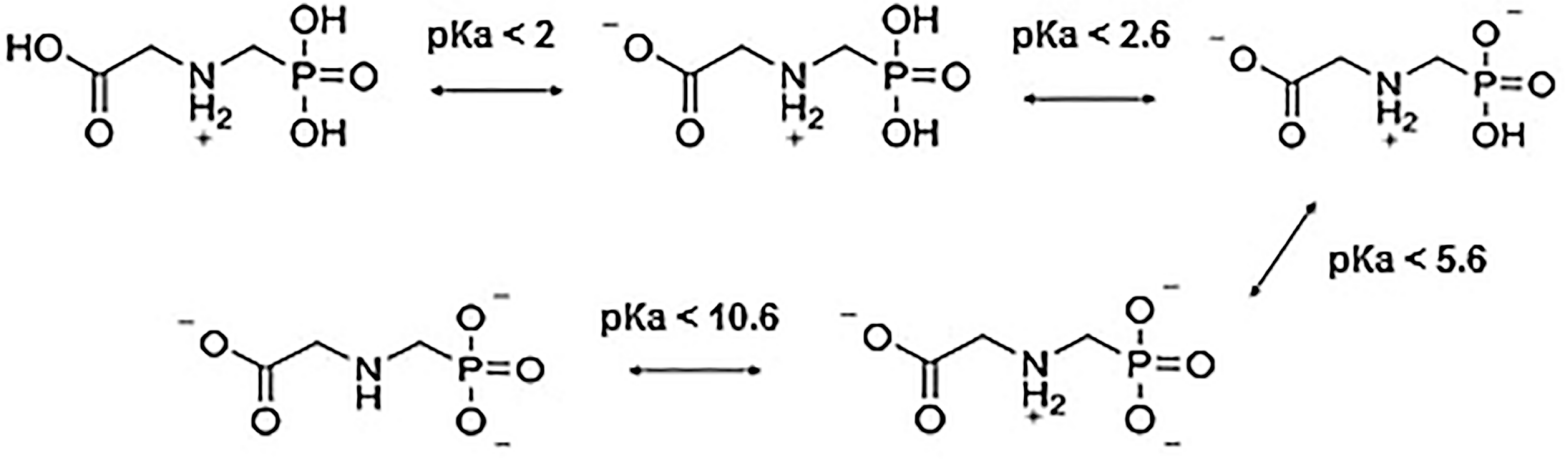
Dissociation of glyphosate according to the acid dissociation constants (p*K*_a_).

The lack of change in removal with pH is considered a positive result since adsorption operations for effluent treatment using the materials will not be susceptible to changes in their removal due to external effects of pH on the effluent. In subsequent studies, pH values adopted for zeolite, sand, activated carbon, furnace slag, and burning ashes were 10, 4, 10, 7 and 7, respectively.

#### 3.2.6 Adsorption of Different Materials with and without Treatment

The materials influence the adsorption process since surface characteristics determine their adsorption capacity. In this study, 5 different materials were tested in 7 different conditions (they include the 6 types of treatments and the untreated condition, i.e., pure material). Results of removal from different materials are listed in Figure 8.

**Figure 8:**
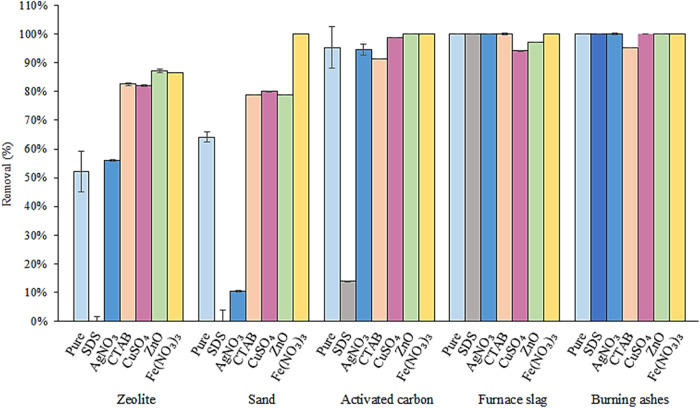
Removal (%) resulting from treatments. Contact time of 24 h, at 110 rpm, m/v ratio of the solution of 25 g·L^–1^ (1 g of adsorbent/40 mL of glyphosate solution) at 25 °C and initial glyphosate concentration of 1 mg·L^–1^.

In most treatments (except for the treatment of sand with Fe­(NO_3_)_3_), removal results were lower for zeolite and sand. This fact may be related to the composition of both materials, since zeolite and sand are compounds that mainly contain silicon and aluminum oxides, unlike activated carbon and burning ashes, which contain carbonaceous compounds, and furnace slag, which has a more complex mixture that comprises heavy metals.^43–47,28^

Regarding the treatments, the one with SDS exhibited the lowest removal capacity by comparison with the other conditions for zeolite (0%), sand (0%), and activated carbon (13.71%). It may be related to the negative charge of SDS, since glyphosate also has a predominantly negative charge, as shown in Figure 7. Concerning furnace slag, treatments with ZnO and CuSO_4_ exhibited removals of 97.20% and 94.19%, respectively, while the others (pure furnace slag, SDS, AgNO_3_, CTAB, and Fe­(NO_3_)_3_) reached 100%. In the case of tobacco, the treatment with CTAB exhibited 95.28% removal while the others reached 100%. A possible explanation for the small change (>6% difference in values) in removal results of furnace slag and burning ashes is that the treatment applied to the materials may not have caused changes in their surfaces. Results of furnace slag and burning ashes also indicate that, regardless of the treatment, interaction between the material and glyphosate remains high and, therefore, application of materials may be done without any treatment (pure materials), aiming at simplicity and economy in the process.

Results of this study indicate that activated carbon treated with CuSO_4_, ZnO, and Fe­(NO_3_)_3_ had higher removal percentages (98.68%, 100%, and 100%, respectively) than treatments with AgNO_3_, CTAB, and SDS (94.61%, 91.53% and 13.71%, respectively). Another study showed that the modification of activated carbon with ZnO increases glyphosate removal.^23^

Regarding treatments applied to zeolite, the glyphosate removal capacity with the modification by CuSO_4_ is more efficient than that of pure zeolite, which is in agreement with another study in which modified zeolite 4A with CuSO_4_ led to a significant increase in adsorption efficiency.^36^ This modification appears to improve the interaction between zeolite and glyphosate and enhance the removal of the contaminant from the solution.

Currently, there are no studies that focus on the evaluation of glyphosate adsorption in foundry waste, such as furnace slag, burning ashes, and sand. However, some studies state that materials containing iron and aluminum oxides (common in foundry waste) may influence glyphosate adsorption.^48^

Results of this study, together with the study of the effect of pH, indicated that burning ashes exhibited the highest level of glyphosate adsorption among the materials under study. Although CTAB showed lower removal than the other treatments (95.28%), results still indicate good efficacy under all conditions and that burning ashes, in general, proved to be efficient in removing the contaminant.

#### 3.2.7 Studies of Dosage (Untreated Burning Ashes)

Variation in the amounts of adsorbent allows not only to observe the relation between the mass and the percentage of pollutant removal but also to define a reasonable value of mass/volume ratio to be applied to the process. Figure 9 shows the removal results of untreated burning ashes (material selected by the previous study).

**Figure 9:**
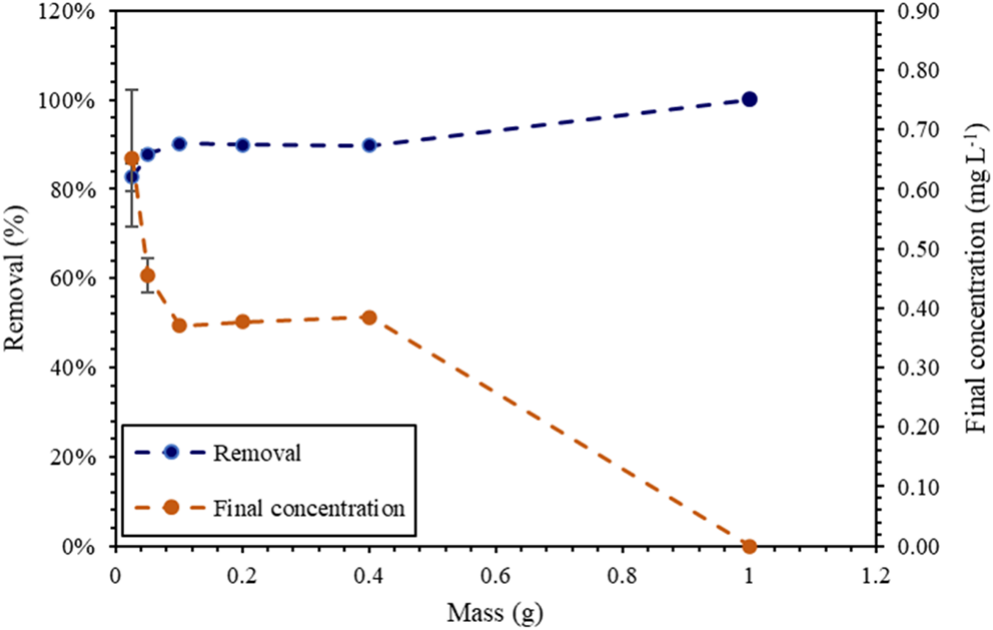
Final concentration and percentage of glyphosate removal from the burning ashes mass. Initial glyphosate concentration of 5 mg L^–1^, pH 7, and contact time of 24 h. Masses: 0.025; 0.05; 0.1; 0.2; 0.4; and 1 g.

An increase in material mass from 0.025 to 0.05 g (2×) resulted in an increase of approximately 5% in removal (82.63% to 87.91%), while subsequent increases in mass, from 0.05 to 0.4 g, did not result in increases above 2%. Increase from 0.4 to 1.0 g allowed an increase of 10%, reaching 100% removal (considering the quantification limit of the method of 0.025 mg·L^–1^). This behavior suggests that, in adsorption, initial gains in removal are limited by the adsorbate concentration or by the equilibrium of the system, while subsequent increases are related to an increase in the availability of active sites of the material. In studies of kinetics and equilibrium, the smallest possible amount of material (0.05 g) was used to meet the maximum limits permitted for glyphosate (0.5 mg·L^–1^) in the Brazilian legislation on water quality for human consumption.^9^

#### 3.2.8 Kinetics (Untreated Burning Ashes)

Figure 10 shows the kinetic results of glyphosate adsorption by burning ashes.

**Figure 10:**
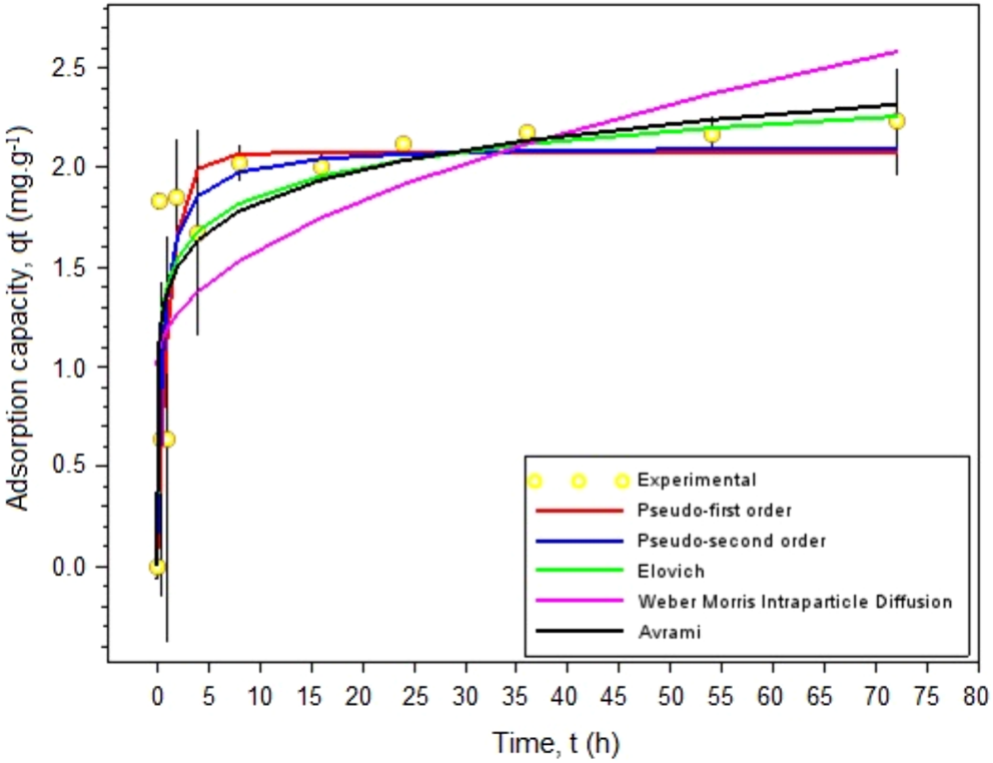
Adsorption capacity of burning ashes (mg·g^–1^) onto glyphosate as a function of time (h) and kinetic modeling. Contact times of 0.17, 0.5, 1, 2, 4, 8, 16, 24, 36, 54, and 72 h under agitation at 145 rpm, solution m/v ratio of 1.25 g·L^–1^ (0.05 g burning ashes/0 mL glyphosate solution) at 25 °C and initial glyphosate concentration of 5 mg·L^–1^.

Adsorption capacity of glyphosate increases rapidly in the first 10 h and reaches 2.02 mg·g^–1^ in the first 8 h. To determine the equilibrium time, removal percentages resulting from each contact time were used, and increase in the removal value was calculated for each increase in time (Table 4). Thus, it may be stated that, considering values below 5% not significant, the system reaches equilibrium in 8 h. After this time, the system begins to show stabilization.

**Table 4:** Adsorption Kinetics: Adsorption Capacity, Removal, and Difference in Removal Over Time

time (h)	adsorption capacity (*q*_t_, in mg·g^–1^)	removal (%)	difference in removal
0,17	1.83	74.42	-
0,5	0.64	25.86	–48.56
1	0.64	25.92	0.06
2	1.85	75.16	49.24
4	1.67	67.87	–7.29
8	2.02	82.21	14.34
16	2.00	81.44	–0.77
24	2.12	86.00	4.56
36	2.18	89.00	3.00
54	2.16	93.94	4.94
72	2.23	96.37	2.43

Concerning glyphosate adsorption, a study reported an equilibrium time of 30 min using activated carbon and 45 min using activated carbon loaded with silver nanoparticles,^23^ while another study observed an equilibrium time of 6 h using polymer-based spherical activated carbon (PBSAC).^49^ A study of zeolite 4A modified with CuSO_4_ observed an equilibrium time of 30 min,^36^ and a time of 3 h was observed for NaY zeolite and Fe-loaded NaY zeolite.^50^ These studies suggest that although glyphosate and burning ashes exhibit affinity, the mass transfer process is slower by comparison with activated carbon and zeolite adsorbents. It may result from some factors, such as intraparticle diffusion, pore size, and chemical interactions among compounds. In addition, initial concentrations of the adsorbates may influence the speed of the process.

The pseudo-first-order, pseudo-second-order, Elovich, Weber-Morris intraparticle diffusion, and Avrami kinetic models were applied to characterize adsorption kinetics (Table 5). According to the values of the coefficient of determination (*r* ^2^) and chi-square (χ^2^), the model that best adjusted the process was the Avrami one. The Avrami kinetic model describes fractional-order reactions, suggesting that interaction processes undergo changes in the mechanism and reaction rate during the time under analysis.^51^ The change in the mechanism may be related to the heterogeneity in the adsorption energy of glyphosate on the surface of the material, a characteristic observed by the equilibrium study, which had a better fit with the Freundlich model than with the Langmuir model.

**Table 5:** Comparison of Kinetic Models: Parameters, Coefficient of Determination (*r* ^*2*^), and Chi-Square (χ^2^)

kinetic models	equation parameters		*r* ^2^	χ^2^
pseudo-first order	*q*_e_ (mg·g^–1^)	2.07	0.53	9.65
	*k*_1_ (min^–1^)	0.81		
pseudo-second order	*q*_e_ (mg·g^–1^)	2.11	0.58	4.27
	*k*_2_ (g·mg^–1^·min^–1^)	0.85		
Elovich	α (mg·g^–1^·min^–1^)	231.24	0.72	1.41
	β (g mg^–1^)	5.02		
Weber-Morris	*k*_dif_ (mg·g^–1^·min^–0,5^)	0.18	0.50	2.62
	*C* (mg·g^–1^)	1.01		
Avrami	*q*_e_ (mg·g^–1^)	7.48	0.73	1.34
	*k*_av_ (min^–1^)	0.20		
	*n*_av_	0.14		

To evaluate the parameters of the adjusted models, they were compared with previously published studies of glyphosate adsorption on different materials, as shown in Table 6. It may be observed that the kinetic models demonstrated significant variations in the parameters. The pseudo-first-order kinetics exhibited *q*_e_ values ranging from 0.7669 mg·g^–1^ for coconut shell activated carbon to 138.73 mg·g^–1^ for activated carbon with nanosilver; tobacco exhibited a value close to those of synthetic materials, such as NaY zeolite and coconut shell activated carbon. The pseudo-second-order kinetics showed a better fit in most cases, with *q*_e_ ranging from 0.7669 mg·g^–1^ (coconut shell activated carbon) to 130.12 mg·g^–1^ (activated carbon with nanosilver). Although the pseudo-first order and pseudo-second order models did not result in the best fits, *k*_1_ and *k*_2_ parameters are above the comparative studies, with the exception of the study that used activated carbon with nanosilver as a glyphosate adsorbent, indicating high-speed kinetics.

**Table 6:** Modeling Parameters of Adsorption Kinetics of Different Adsorbent Materials in Glyphosate Removal

	pseudo-first order			pseudo-second order			Elovich			Weber-Morris intraparticle diffusion			
kinetic models	*Q*_e_ (mg·g^–1^)	*K*_l_ (min^–1^)	*r* ^*2*^	*Q*_e_ (mg·g^–1^)	*K*_2_ (g·mg^–1^·min^–1^)	*r* ^*2*^	α (mg·g^–1^·min^–1^)	β (g·mg^–1^)	*r* ^*2*^	*k*_dif_ (mg·g^–1^·min^–0,5^)	*C* (mg·g^–1^)	*r* ^*2*^	refs
RClay- biochar	24.749	0.025	0.987	41.010	0.002	0.999							^20^
zeolite 4A-CuSO_4_	30.33	0.05187	0.9755	32.50	0.00329	0.9924							^36^
activated carbon with nanosilver	138.73	0.038	0.5767	130.12	0.934	0.9983				8.034	45.667	0.8671	^23^
rice husk biochar	31.555	0.0318	0.9723	35.7057	0.0013	0.8984	7.9974	0.1732	0.7922				^16^
woody biochar	19.812	0.038	0.940	22.774	0.002	0.983	2.170	0.215	0.873				^18^
carbon nanotube	20.07	0.03067	0.9982	24.49	0.001297	0.996	0.1389	0.08848	0.7803				^56^
zeolite NaY	1.47	0.018	0.8581	2.80	0.0238	0.9942	2.81	2.55	0.9696	0.1013	1.5125	0.9853	^49^
coconut shell activated carbon	0.7669	0.1114	0.9897	0.7669	0.5812	0.9976							^17^
woody biochar	0.8664	0.1471	0.9782	0.8664	0.1581	0.9997							^17^
burning ashes	2.07	0.81	0.53	2.11	0.85	0.58	231.24	5.02	0.72	0.18	1.01	0.50	this study

The Elovich model was applied to some adsorbents and exhibited distinct α and β parameters, with α values ranging from 0.1389 mg·g^–1^·min^–1^ for carbon nanotube to 7.9974 mg·g^–1^·min^–1^ for rice husk biochar and β ranging from 0.08848 g·mg^–1^ for carbon nanotube to 2.55 g·mg^–1^ for NaY zeolite. Regarding tobacco, α and β were 231.24 mg·g^–1^·min^–1^ and 5.02 g·mg^–1^, respectively; that is, both were higher than values found by the other studies. Values of α and β represent the initial adsorption rate and the desorption rate, respectively.^52^ Therefore, it may be stated that kinetics of glyphosate adsorption onto burning ashes is high (speed) at the concentration under study. It corroborates the values of the pseudo-first order and pseudo-second order parameters, although these models were not the best fit ones.

Regarding the Weber-Morris model, the *k*_dif_ value ranged between 0.1013 mg·g^–1^·min^–0.5^ for NaY zeolite and 8.034 mg·g^–1^·min^–0.5^ for activated carbon with nanosilver, while tobacco exhibited 0.18 mg·g^–1^·min^–0.5^. The value of constant *C* was between 1.5 and 45 mg·g^–1^ in the other studies, and for tobacco, it was 1.01 mg·g^–1^. The *C* value is related to the thickness of the solvent film around the adsorbent: the higher the *C* is, the greater the effect of intrafilm diffusion on the adsorption rate; values close to zero suggest control by intrapore diffusion, where surface porosity is decisive.^53^ The *k*_dif_ parameter is the intraparticle diffusion coefficient.^23^

Concerning studies of glyphosate removal, the Avrami model was only adjusted by the study carried out with carbon nanotubes, with values of *q*_e_, *k*_av_, and *n*_av_ equivalent to 20.06 mg·g^–1^, 0.2043 min^–1^, and 0.1503, respectively (these constants of the Avrami model were omitted in Table 6 to improve its visualization, as it is a single study). Values of *q*_e_, *k*_av_, and nav for tobacco were 7.48 mg·g^–1^, 0.20 min^–1^, and 0.14, respectively; *n*_av_ is the dimensionless Avrami number, and *k*_av_ is the Avrami rate constant.^54^ The higher the *k*_av_ value, the faster the adsorption process reaches equilibrium.^55^ The *n*_av_ value may provide information about the adsorption mechanism, such as the trend toward surface change over time and the expansion of the number of adsorption sites.

#### 3.2.9 Adsorption Isotherms (Untreated Burning Ashes)

Figure 11 shows experimental results of the study of glyphosate adsorption equilibrium by burning ashes, as well as the Langmuir, Freundlich, BET, and Temkin isotherm models applied to characterize the isotherm. Table 7 shows the removal values resulting from the concentration range under study. Model adjustment parameters, *r* ^*2*^ and *X* ^*2*^, are shown in Table 8.

**Figure 11:**
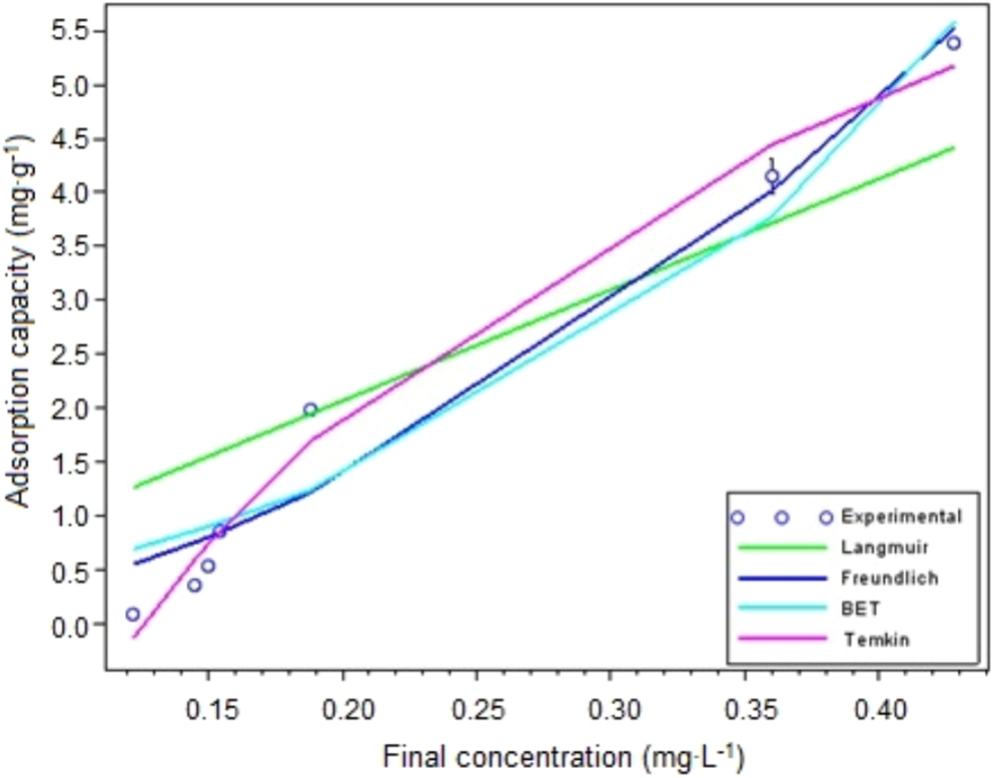
Experimental kinetic data and fitted rate models. Contact time 24 h, agitation at 145 rpm, solution m/v ratio of 1.25 g·L^–1^ (0.05 g burning ashes/40 mL glyphosate solution) at 25 °C, and initial glyphosate concentrations of 0.25; 0.5; 1.0; 1.5; 2.5; 5.0 and 7.5 mg·L^–1^.

**Table 7:** Experimental Values

initial concentration (mg·L^–1^)	0.25	0.50	1.00	1.50	2.50	5.00	7.500
final concentration (mg·L^–1^)	0.122	0.145	0.159	0.154	0.188	0.360	0.428
*q*_e_ (mg·L^–1^)	0.085	0.347	0.524	0.849	1.987	4.150	5.391
removal (%)	46.58	75.00	80.51	87.30	92.97	93.57	94.03

**Table 8:** Comparison of Isotherm Models: Parameters, Coefficient of Determination (*r* ^*2*^), and Chi-Square (χ^2^)

isotherm models	equation parameters		*r* ^*2*^	χ^2^
Langmuir	*q*_emax_ (mg·g^–1^)	259.98	0.78	3.38
	*K*_l_ (L·mg^–1^)	0.04		
Freundlich	*K*_f_ (mg·g^–1^ (mg·L^–1^)^−1/*n*^)	26.38	0.96	1.26
	*n*	7.00		
BET	*q*_BET_ (mg·g^–1^)	167.27	0.94	1.55
	*K*_1_ (L·mg^–1^)	0.03		
	*K*_2_ (L·mg^–1^)	0.99		
Temkin	*B*	4.24	0.99	0.24
	*A* (L·mg^–1^)	7.93		

It may be seen that the adsorption capacity shows a continuous increase in the concentration range under study, without any behavior or tendency toward saturation. Another aspect is the shape of the graph, which apparently changes, from concave up to approximately 0.2 mg·L^–1^ to convex up to the end of the concentration range under evaluation (i. e., an “S” shape).

According to values in Table 7, the maximum adsorption capacity of glyphosate onto burning ashes in the concentration range under evaluation was 5.39 mg·g^–1^, when 94.03% removal was reached.

Considering the fitting parameters of this study (Table 8), it may be stated that the Temkin, Freundlich, and BET isotherm models fitted the data better than the Langmuir isotherm. Calculated values of *r* ^2^ (0.99) and χ^2^ (0.24) show that the Temkin model fitted glyphosate adsorption on tobacco better. The fitting of the Temkin isotherm model suggests that glyphosate adsorption was controlled by electrostatic interactions and chemical adsorption.

Maximum adsorption capacity, *q*_emax_, was calculated by the Langmuir model and resulted in 259.98 mg·g^–1^. However, the Langmuir model reached the lowest *r* ^2^ value and the highest χ^2^ value, which suggests that the adsorbent has neither a homogeneous surface nor a monolayer compound. The comparison among parameters of the isotherm models used by this study and the values found in the literature reveals significant differences in the maximum adsorption capacity (*q*_emax_) and in the coefficients of the equations. In the Langmuir model, this study reached a *q*_emax_ of 259.98 mg·g^–1^ and a *K*_l_ coefficient of 0.04 L·mg^–1^; *r* ^2^ was 0.78. These values contrast with those reported in the literature, in which *q*_emax_ values did not exceed 150 mg·g^–1^ but *r* ^2^ was higher than that of this study, except for coconut shell activated carbon and woody biochar (*r* ^2^ of 0.061 and 0.124, respectively). Although the Langmuir *q*_m_ coefficient exhibited a high value, it is important to highlight that experiments at higher concentrations are needed to validate this observation.

By fitting the Freundlich model, this study found a *K*_f_ value of 26.38 mg·g^–1^(mg·L^–1^)^−1/n^ and n value of 7.00; *r* ^2^ was 0.96. *K*_f_ values refer to the adsorption capacity of adsorbents.^23^ By comparison with the literature, *K*_f_ values varied widely among the different adsorbents, such as activated carbon with nanosilver, whose *K*_f_ was 188.96 mg·g^–1^(mg·L^–1^)^−1/*n*^, and activated carbon from coconut shell and woody biochar, whose *K*_f_ values were 0.0941 mg·g^–1^(mg·L^–1^)^−1/*n*^ and 0.2558 mg·g^–1^(mg·L^–1^)^−1/*n*^, respectively. This study exhibited a *K*_f_ value similar to the ones of rice husk biochar (21.66 mg·g^–1^(mg·L^–1^)^−1/*n*^) and water treatment residue (30.116 mg·g^–1^(mg·L^–1^)^−1/*n*^), but their *n* parameters diverged (2.45 and 0.264).

Values of the *n* parameter, which reflect adsorption intensity, ranged between 0.264 and 8.227 in the literature, and the material of this study was within this range. The value of the Freundlich constant (*n*) above 1 and below 10 found by this study (*n* = 7.00, shown in Table 8) indicates favorable conditions and heterogeneity in the glyphosate adsorption process.^23,36^ Values mentioned in the literature and references to every study are shown in Table 9.

**Table 9:** Modeling Parameters of Adsorption Isotherms of Different Adsorbent Materials in Glyphosate Removal

	Langmuir			Freundlich			Temkin			
adsorbent material	*Q*_emax_ (mg·g^–1^)	*K*_l_ (L·mg^–1^)	*r* ^*2*^	*K*_f_ (mg·g^–1^ (mg·L^–1^)^−1/*n*^)	*n*	*r* ^2^	*B* (J·mol^–1^)	*A* (L·g^–1^)	*r* ^*2*^	refs
Clay-biochar	2.712	22.148	0.993	5.913	2.045	0.928	13.812	0.396	0.983	^20^
zeolite 4A-CuSO_4_	121.70	0.00933	0.997	2.114	1.349	0.996	116.977	0.1242	0.983	^36^
activated carbon with nanosilver	149.25	0.85	0.899	188.96	8.227	0.999	95.38	5.781	0.867	^23^
rice husk biochar	123.03	0.0892	0.935	21.66	2.45	0.983	-	-	-	^16^
woody biochar	44.01	0.088	0.91	7.27	0.406	0.96	1.788	1.0	0.92	^18^
water treatment residue	113.636	0.096	0.999	30.116	0.264	0.885	18.698	2.200	0.9534	^57^
coconut shell activated carbon	1.0549	0.0861	0.061	0.0941	1.0873	0.811	-	-	-	^17^
woody biochar	1.1645	0.0451	0.124	0.2558	0.9772	0.9567	-	-	-	^17^
burning ashes	259.98	0.04	0.78	26.38	7.00	0.96	4.24	7.93	0.99	this study

In the BET model, the maximum adsorption capacity, *q*_BET_, was calculated and resulted in 167.27 mg·g^–1^. *K*_1_ and *K*_2_ coefficients were 0.03 and 0.99, respectively, and the *r* ^2^ fit was 0.94. However, since BET values of glyphosate adsorption are scarce in the literature, it is difficult to make a more detailed comparison. Although BET and Langmuir did not exhibit the best model adjustments, values of the *q*_BET_ and q_emax_ parameters indicate high adsorption capacity by comparison with the study that used only glyphosate.

In the Temkin model, parameters *B* and *A* were 4.24 J·mol^–1^ and 7.93 L·g^–1^, respectively, with an adjustment (*r* ^2^) of 0.99. Considering that a positive value of B indicates an endothermic process, which implies the need to supply energy, lower values are preferable. Thus, in the comparison among the reported data (Table 9), burning ashes exhibited the second-best value, following woody biochar. A ranged between 0.1242 and 5.781 L·g^–1^. These results indicate that the performance of the material used by this study was superior to that of most adsorbents described in the literature. Therefore, it has promising potential to remove the contaminants under investigation.

## 4 Conclusions

Results of this study demonstrate that adsorption by commercial materials and industrial waste is a viable alternative for the removal of glyphosate from aqueous solutions. Among materials under study, burning ashes exhibited the highest adsorption, since herbicide removal was above 87% under all conditions applied herein. The highest removal percentages after different treatments of materials under study were: 87.26% (zeolite removed by ZnO), 100% (sand by Fe­(NO_3_)_3_), 100% (activated carbon by ZnO and Fe­(NO_3_)_3_), 100% (furnace slag removed by all treatments except CuSO_4_ and ZnO) and 100% (burning ashes by all treatments except CTAB). Besides, adsorption onto burning ashes did not change with pH, resulting in 100% removal at pH of 4, 7, and 10 (with a minimum detection limit of 0.025 mg·L^–1^). However, the other materials showed changes in adsorption when the pH was altered.

In the dosage study, burning ashes exhibited 100% removal at the dose of 25 g·L^–1^ and 87.91% at the dose of 1.25 g·L^–1^. The kinetic analysis indicated that adsorption equilibrium is reached within 8 h, and the Avrami model was the best fit to the data. The study of equilibrium revealed the maximum capacity of 5.39 mg·g^–1^ and the Temkin isotherm model had the best fit. The analytical method based on derivatization, followed by LC-MS analysis, proved suitable for quantifying glyphosate, ensuring reliability to the results. Thus, this study contributes to the development of sustainable strategies for treating water contaminated by glyphosate with the use of low-cost, accessible materials.
